# Serum ACE2 activity correlates with infarct size and left ventricular dysfunction during acute myocardial infarction

**DOI:** 10.1186/1532-429X-13-S1-P142

**Published:** 2011-02-02

**Authors:** José T Ortiz Pérez, Marta Riera, Xavier Bosch Genover, Teresa María De Caralt, Rosario Jesús Perea, Julio Pascual, María José Soler Romeo

**Affiliations:** 1 Thorax Institute. Hospital Clinic Barcelona, Barcelona, Spain; 2 Hospital del Mar. Fundación IMIM. Parc de Salut Mar, Barcelona, Spain; 3 Radiology Department. Hospital Clinic Barcelona, Barcelona, Spain

## Background

Angiotensin converting enzyme-2 (ACE2) is an analogue of the ACE that cleaves Angiotensin II to Angiotensin 1-7, a peptide with vasodilatory properties. Previous studies showed increased ACE2 activity among patients with chronic heart failure. ACE2 activity has been shown to correlate with the NYHA functional class in this setting, suggesting a compensatory role in advanced heart failure. The relationship between ACE2 activity and myocardial function in patients with ST-elevation acute myocardial infarction (STEMI) remains unknown.

## Aim

We sought to explore the relationship between ACE2 activity, infarct size and acute left ventricular (LV) systolic function following STEMI.

## Methods

We prospectively included 44 patients with their first STEMI reperfused by primary percutaneous intervention. Baseline blood samples were drawn between 12 and 24 hs from admission and at 7 days. A standard cine and contrast enhanced-cardiac magnetic resonance study was performed during admission. LV end-diastolic (LVED) and end-systolic (LVES) indexes, ejection fraction (EF) and infarct size (expressed as % of the LV mass), were computed by manual plannimetry on sequential short axis cine and delayed enhanced images, respectively. Serum ACE2 activity was measured by fluorometric assay using a specific substrate.

## Results

Baseline ACE2 activity did not correlate with LV volume indexes, EF, infarct size and BNP. By paired analysis, ACE2 activity significantly increased from baseline to 7 days in STEMI patients (104±66 to 126±84 RFU/µl/hr, p<0.02). Furthermore, at 7 days a significant direct correlation was observed between ACE2 activity and all, LVES index (r=0.41, p<0.02), infarct size (r=0.34, p<0.05) and baseline BNP levels (r=0.36, p<0.05). Additionally, a significant inverse correlation between ACE2 activity and EF was observed (r=-0.39, p<0.01). See figure.

## Conclusion

ACE2 serum activity rises during the first week following STEMI and the increase is related to infarct size and underlying indicators of LV systolic dysfunction. As suggested in chronic heart failure stages, ACE2 activation may reflect a compensatory mechanism to limit left ventricular dysfunction and could potentially indicate a new target for therapy following STEMI reperfusion.

